# The ileal fungal microbiota is altered in Crohn's disease and is associated with the disease course

**DOI:** 10.3389/fmed.2022.868812

**Published:** 2022-09-27

**Authors:** Maya Olaisen, Mathias L. Richard, Vidar Beisvåg, Atle van Beelen Granlund, Elin S. Røyset, Olivier Rué, Tom Christian Martinsen, Arne Kristian Sandvik, Harry Sokol, Reidar Fossmark

**Affiliations:** ^1^Department of Clinical and Molecular Medicine, Faculty of Medicine and Health Sciences, Norwegian University of Science and Technology, Trondheim, Norway; ^2^Department of Gastroenterology and Hepatology, St. Olav's Hospital - Trondheim University Hospital, Trondheim, Norway; ^3^INRAE, AgroParisTech, Micalis Institute, Université Paris-Saclay, Jouy-en-Josas, France; ^4^Paris Center for Microbiome Medicine, Fédération Hospitalo-Universitaire, Paris, France; ^5^Central Administration, St. Olav's Hospital - Trondheim University Hospital, Trondheim, Norway; ^6^Centre of Molecular Inflammation Research, Faculty of Medicine and Health Sciences, Norwegian University of Science and Technology, Trondheim, Norway; ^7^Department of Pathology, St. Olav's Hospital - Trondheim University Hospital, Trondheim, Norway; ^8^INRAE, MaIAGE, Université Paris-Saclay, Jouy-en-Josas, France; ^9^INRAE, BioinfOmics, MIGALE Bioinformatics Facility, Université Paris-Saclay, Jouy-en-Josas, France; ^10^Gastroenterology Department, INSERM, Centre de Recherche Saint-Antoine, CRSA, AP-HP, Saint Antoine Hospital, Sorbonne Université, Paris, France

**Keywords:** Crohn's disease, inflammatory bowel disease, mycobiota, fungal microbiota, fungi

## Abstract

**Introduction:**

Fungal microbiota's involvement in the pathogenesis of Crohn's disease (CD) is incompletely understood. The terminal ileum is a predilection site both for primary involvement and recurrences of CD. We, therefore, assessed the mucosa-associated mycobiota in the inflamed and non-inflamed ileum in patients with CD.

**Methods:**

The mucosa-associated mycobiota was assessed by ITS2 sequencing in a total of 168 biopsies sampled 5 and 15 cm proximal of the ileocecal valve or ileocolic anastomosis in 44 CD patients and 40 healthy controls (HC). CD patients with terminal ileitis, with endoscopic inflammation at 5 cm and normal mucosa at 15 cm and no history of upper CD involvement, were analyzed separately. The need for additional CD treatment the year following biopsy collection was recorded.

**Results:**

CD patients had reduced mycobiota evenness, increased Basidiomycota/Ascomycota ratio, and reduced abundance of Chytridiomycota compared to HC. The mycobiota of CD patients were characterized by an expansion of *Malassezia* and a depletion of *Saccharomyces*, along with increased abundances of *Candida albicans* and *Malassezia restricta*. *Malassezia* was associated with the need for treatment escalation during follow-up. Current anti-TNF treatment was associated with lower abundances of Basidiomycota. The alpha diversity of the inflamed and proximal non-inflamed mucosa within the same patients was similar. However, the inflamed mucosa had a more dysbiotic composition with increased abundances of *Candida sake* and reduced abundances of *Exophiala equina* and *Debaryomyces hansenii*.

**Conclusions:**

The ileal mucosa-associated mycobiota in CD patients is altered compared to HC. The mycobiota in the inflamed and proximal non-inflamed ileum within the same patients harbor structural differences which may play a role in the CD pathogenesis. Increased abundance of *Malassezia* was associated with an unfavorable disease course.

## Introduction

Crohn's disease (CD) is a chronic inflammatory bowel disease (IBD) characterized by transmural and segmental inflammation of the gastrointestinal tract. Currently, CD is thought to develop in genetically susceptible individuals exposed to environmental factors and gut microbiota, causing an aberrant immune response that leads to inflammation and subsequent tissue damage (1). The presence of a luminal factor causing inflammation was early demonstrated as an ileostomy diverting the intestinal contents has a well-known protective effect, whereas reestablishment of bowel continuity or infusion of fecal content triggers recurrence (2, 3). Disease recurrence typically manifests at and immediately proximal to an anastomosis (4, 5) and studies of the terminal and neo-terminal ileum are therefore of particular interest to understand the pathogenesis of CD. The ileal bacterial mucosa-associated microbiota at the time of ileocecal resection (ICR) and postoperatively has been associated with the risk of disease recurrence (6–8).

Several findings also suggest that the mycobiota is involved in CD pathogenesis (9). Anti-*Saccharomyces cerevisiae* antibodies (ASCA) were early proposed as a diagnostic biomarker of CD (10). Genome-wide association studies (GWAS) have later identified *CARD9* single-nucleotide polymorphism (rs4077515 creating substitution p.S12N) to be associated with CD (11, 12). Identification of intestinal fungi through C-lectin receptors depends on *CARD9* in the signaling pathway to stimulate a pro-inflammatory response to commensal fungi (13, 14), and a defect in *CARD9* is associated with susceptibility to fungal infections and a lower number of Th-17 cells in humans (13). In addition, Toll-like receptor 4 polymorphisms associated with both CD and UC also predispose to systemic *Candida* infections in humans (15). A proportion of IBD patients have genetic polymorphisms that increase susceptibility to fungal infections, also the fungal load and richness are elevated in CD patients (16, 17).

Although the majority of studies have analyzed the fecal mycobiota, the mucosa-associated and fecal bacterial microbiotas are different (18–20), and the mucosa-associated microbiota is by many considered more relevant to the pathogenesis of CD (21). Only a few studies have described the mucosa-associated mycobiota in CD patients (14, 16, 22). The mucosa-associated mycobiota in CD is characterized by a skewed Ascomycota to Basidiomycota ratio, increased abundances of Basidiomycota, and decreased abundances of Ascomycota phyla in CD compared to controls (14, 22). Inflamed tissue in CD patients has a 40-fold higher load of fungi compared to healthy controls (HC) and increased abundances of Xylariales order and Sordariomycetes class (16). Water-lavage samples obtained from CD patients during colonoscopy have increased abundances of *Malassezia, Cladosporium*, and *Aureobasidium* and decreased abundances of *Fusarium* compared to HC (14). Notably, *Malassezia* was found to be overrepresented in patients carrying a CARD9 allele which is associated with an increased risk of CD (14). CD has also been associated with increased abundance of Psathyrellaceae and Cortinariaceae families and *Psathyrella* and *Gymnopilus* genera (22) in a cohort of teenagers in Saudi Arabia. More recently*, Debaromyces* have been reported to be abundant in CD ulcerations and could be of importance in the pathogenesis of CD (23). However, the prognostic value of the mycobiota has to the best of our knowledge not been evaluated before.

In the current study, we have assessed the mycobiota of patients with CD and HC, focusing on differences between inflamed and proximal non-inflamed ileal mucosa within CD patients and the association between mycobiota and the clinical course during follow-up.

## Materials and methods

### Patients and control subjects

We have previously assessed the bacterial ileal microbiota of the same patient cohort (24). Study participants were recruited from the Department of Gastroenterology, St. Olav's Hospital, Trondheim, Norway between 2017 and 2019. Patients 18–70 years of age with Norwegian ethnicity and referred to ileocolonoscopy were invited to participate if they were eligible. Inclusion criteria were an established diagnosis of CD based on clinical, endoscopic, and histological criteria or patients with CD symptoms where the diagnosis was confirmed after both endoscopic and histologic evaluation. CD characteristics were registered according to the Montreal classification (25). Age- and sex-matched subjects referred to colonoscopy due to rectal bleeding or screening for the disease were included as healthy controls (HC) if the ileocolonoscopy and histologic evaluation of biopsies were normal. Exclusion criteria were as described by Olaisen et al. (24), that is, use of antibacterial or antifungal treatment for the past 2 months or comorbidity with diabetes mellitus, celiac disease, or liver diseases including primary sclerosing cholangitis and primary biliary cholangitis. Additional exclusion criteria for HCs were previous gastrointestinal surgery, gastrointestinal polyps, cancer, diverticulitis, or irritable bowel disease fulfilling the ROME IV criteria (26). Information about the initiation of treatment escalation against CD the following year after biopsy collection was extracted from the medical records and electronic registry of prescriptions. CD treatment escalation was defined as the initiation of new medication, change within drug class or increased dose of systemic glucocorticoids (including budesonide), immunomodulators (azathioprine and methotrexate), biologics, or surgery, whichever occurred first.

### Endoscopic procedure

The ileum was reached during endoscopy using either a colonoscope (Olympus Exera II GIF HQ190 or PH190L, Olympus Europa GmbH, Hamburg, Germany) or a single-balloon enteroscope (Olympus SIF-Q180). A total of six ileal pinch biopsies were collected from each study participant, three biopsies from approximately 5 and 15 cm proximal of the ileocecal valve or ileocolic anastomosis, respectively. In CD patients with terminal ileitis, the 5-cm samples were taken from an endoscopically inflamed area and 15-cm samples from normal-appearing mucosa. In CD patients categorized as having active disease, both biopsy locations (5 and 15 cm) were endoscopically inflamed. For CD patients in remission and the HC group, both biopsy locations (5 and 15 cm) appeared endoscopically normal. Endoscopic inflammation was evaluated using Rutgeerts score (27), whether the patients had been operated on by ICR or not, with inflammation defined as Rutgeerts score ≥1. One pair of mucosal pinch biopsies from the 5- and 15-cm locations were put on formalin for histological grading of inflammation. The two remaining biopsy pairs were put directly on liquid N_2_ and stored on N_2_ until subsequent bacterial or fungal DNA isolation and sequencing of the bacterial (24) and fungal microbiota, respectively.

### Histological evaluation of biopsies

Formalin-fixed biopsies were stained with hematoxylin and eosin (H&E). Histological examination was performed blinded for phenotype by an experienced pathologist and scored according to the Global Histologic Disease Activity Score (GHAS) and Robarts score (3, 28, 29). A validated histological scoring index for the evaluation of disease activity in CD is lacking, and the reciprocity between histological scoring and disease activity measures is poor (29, 30). However, histological evaluation blinded for phenotype verified all biopsies from HC as histologically normal.

### DNA isolation

The fungal cell wall is particularly robust and is known to be hard to lyse (31, 32). A DNA isolation protocol specially designed to lyse the fungal cell wall, with both a chemical and mechanical lysis step, was therefore chosen. DNA from two mucosal biopsies (at 5 and 15 cm locations) was isolated according to a previously described protocol (33) with the following adjustments; bead beating was performed with Precellys 24 tissue homogenizer (Bertin Technologies, Montigny-le-Bretonneux, France) at 6,500 rpm for 60 s two times. Centrifugation steps were performed at 21,000 g, otherwise, the original protocol was followed (33). The DNA samples were quantified using Qubit (Thermo Fisher Scientific, Waltham, MA).

### ITS2 sequencing

ITS2 metagenomic sequencing libraries were prepared according to the “Illumina Metagenomics Sequencing Demonstrated Protocol” (34) with minor adjustments. In brief, 200 ng genomic DNA (extracted from biopsy samples) was used as a template for PCR amplification of the ITS2 region (98°C at 30 s, followed by 34 cycles with 15 s at 98°C, 53°C for 30 s, and 72°C for 45 s, followed by 7 min at 72°C). The ITS2 PCR primers were based on sequences first published by Liguori et al. (16). Illumina adaptor-compatible overhang nucleotide sequences were added to the gene/locus-specific sequences (ITS2 Amplicon PCR Forward Primer = 5′ TCGTCGGCAGCGTCAGATGTGTATAAGAGACAGGTGARTCATCGAATCTTT and ITS2 Amplicon PCR Reverse Primer = 5′ GTCTCGTGGGCTCGGAGATGTGTATAAGAGACAGGATATGCTTAAGTTCAGCGGGT). The PCR products were then cleaned up by using AMPure XP beads (Beckman Coulter, Woerden, Netherlands) to purify ITS2 amplicons away from free primers and primer dimer species. In a second PCR amplification step (9 cycles), dual indices and Illumina sequencing adaptors were added by using the Nextera XT indexing kit (Illumina Inc., San Diego, CA) according to the manufacturer's instructions. A second PCR clean-up step was performed using AMPure XP beads (Beckman Coulter), before the validation of the library by a LabChip GX DNA high sensitivity assay (PerkinElmer, Inc., Waltham, MA). Libraries were normalized and pooled to 12 pM and subjected to clustering on two MiSeq V3 flowcells. Finally, paired-end read sequencing was performed for 2 x 300 cycles on a MiSeq instrument (Illumina, Inc.), according to the manufacturer's instructions. Base calling was done on the MiSeq instrument by RTA v1.18.54. FASTQ files were generated using bcl2fastq2 conversion software v2.17 (Illumina, Inc.).

### Bioinformatics

Sequencing data were processed using the FROGS pipeline (35, 36) for sequence quality control, filtering, and affiliation of taxa with the UNITE ITS database (version 8_2) (37), using the FROGS guidelines for ITS data (http://frogs.toulouse.inra.fr/). Five biopsy samples were removed from the study due to a low number of sequences. This included two 5-cm samples from HC and three 5-cm samples from CD patients. Phyloseq Package for R analysis was used for alpha and beta diversity analyses as well as illustration. Deseq2 package for R analysis was used for differential analysis of OTUs with respect to the different phenotypes (38). The linear discriminant analysis (LDA) effect size (LEfSe) algorithm (39) was used to identify taxa that were specific to phenotype or inflamed vs. proximal non-inflamed mucosa.

### Statistics

IBM SPSS Statistics version 25.0 (IBM Corp., Armonk, NY) was used for statistical analysis apart from analyses of sequencing data. Demographic and clinical characteristics are presented as % (n) for categorical variables, median [interquartile range (IQR)] for skewed distributed variables, and mean value [standard deviation (SD)] for normally distributed variables. Accordingly, the chi-squared test, Mann–Whitney *U* test, or independent *t*-test were used for comparing CD patients with HC. For all statistical analyses, a *p*-value < 0.05 was considered statistically significant.

### Ethical considerations

The study was approved by the Regional Committee for Medical and Health Research Ethics, Central Norway (approval reference, 2016/2164). All study participants provided written informed consent.

## Results

### Patients

Forty-four CD patients and 40 HC were included. Demographic and clinical characteristics are presented in Table 1. CD patients had higher CRP levels compared to HC (*p* = 0.017). The groups were otherwise similar. The bacterial microbiota characteristics in this cohort have been described previously (24). CD characteristics are provided in Table 2. Twenty-two CD patients had terminal ileitis with endoscopic inflammation at the 5-cm location and normal mucosa at the 15-cm location, of which 20 had no history of upper gastrointestinal CD involvement. Of the remaining CD patients, 10 had active disease and 12 were in remission.

**Table 1:** Demographic and clinical characteristics of Crohn's disease (CD) patients and healthy controls (HC).

	**CD**	**HC**	***p*** **-value^a^**
**Number of patients**, ***n***	44	40	
**Male gender**, ***n*** **(%)**	24 (54.5%)	19 (47.5%)	0.52
**Age**, ***years*****, mean (SD)**	42.2 (14.4)	36.6 (12.9)	0.07
**BMI, mean (SD)**	25.8 (4.8)	26.6 (4.7)	0.40
**Acid reflux medication**, ***n*** **(%)**			0.72
PPI	5 (11.4%)	2 (5%)	
H_2_ blockers	0	0	
PPI on demand	0	0	
H_2_ blockers on demand	1 (2.3%)	1 (2.5%)	
**Smoking**, ***n*** **(%)**			0.57
Never smoker	23 (52.3%)	25 (62.5%)	
Active smoker	5 (11.4%)	5 (12.5%)	
Snuff	10 (22.7%)	8 (20%)	
Ex-smoker	6 (13.6%) %)	2 (5%)	
**Laboratory values**			
Hb (g/dL), mean (SD)	14.1 (1.5)	14.5 (1.7)	0.197
Leukocytes (x10^9^/L), median (IQR)	6.4 (2.3)	6.5 (2.3)	0.50
CRP (mg/L), median (IQR)	<5 (4)	<5 (0)	**0.017**

a Comparing CD (*n* = 44) with HC (*n* = 40) using Mann–Whitney *U*-test for skewed distributed continuous variables, independent *t*-test for normal distributed continuous variables, and Chi-square/Fisher exact test for categorical variables.

**Table 2:** Crohn's disease (CD) characteristics, medical treatment, endoscopic evaluation, and surgical history.

**CD characteristics**	**CD (*n =* 44)**
**Disease duration**, ***years (median, IQR)***	10.0 (19.8)
**Subclassification of patients**, ***n*** **(%)**^**a**^	
Terminal ileitis (Inflamed 5-cm + normal 15-cm)	22 (50.0%)
Active disease (Inflamed 5-cm + 15-cm)	10 (22.7%)
Remission (Normal 5 + 15 cm)	12 (27.3%)
**Montreal location**, ***n*** **(%)**	
Terminal ileum (L1)	23 (52.3%)
Ileocolonic (L3)	16 (36.4%)
Ileocolonic + Upper GI (L3 + L4)	5 (11.4%)
**Montreal behavior**, ***n*** **(%)**	
Non-stricturing, non-penetrating (B1)	8 (18.2%)
Non-stricturing, non-penetrating + perianal (B1p)	2 (4.5%)
Stricturing (B2)	15 (34.1%)
Stricturing + perianal (B2p)	6 (13.6%)
Penetrating (B3)	11 (25%)
Penetrating + perianal (B3p)	2 (4.5%)
**Montreal age (age at diagnosis)**, ***n*** **(%)**	
16 years or younger (A1)	12 (27.3%)
17–40 years (A2)	22 (50%)
Over 40 years (A3)	10 (22.7%)
**CD-medication**, ***n*** **(%)**^**b**^	
No medical therapy for CD	18 (40.9%)
Budesonide	7 (15.9%)
Prednisolone	4 (9.1%)
5-ASA	3 (6.8%)
Azathioprine	6 (13.6%)
Methotrexate	3 (6.8%)
Adalimumab	4 (9.1%)
Infliximab	7 (15.9%)
Vedolizumab	1 (2.3%)
Treatment naïve, *n (%)*	6 (13.6%)
Anti-TNF treatment naïve, *n (%)*	23 (52.3%)
**Rutgeerts score**, ***n*** **(%)**	
i0	12 (27.3%)
i1	12 (27.3%)
i2	5 (11.4%)
i3	6 (13.6%)
i4	9 (20.5%)
**Ileocecal resection**	28 (63.6%)

a Based on endoscopic evaluation of inflammation.

b Co-medication: *n* = 8 (18.2) used two CD medications, *n* = 1 (2.3%) used three CD medications.

### Ileal mycobiota in CD patients vs. HC

**Figure 1:**
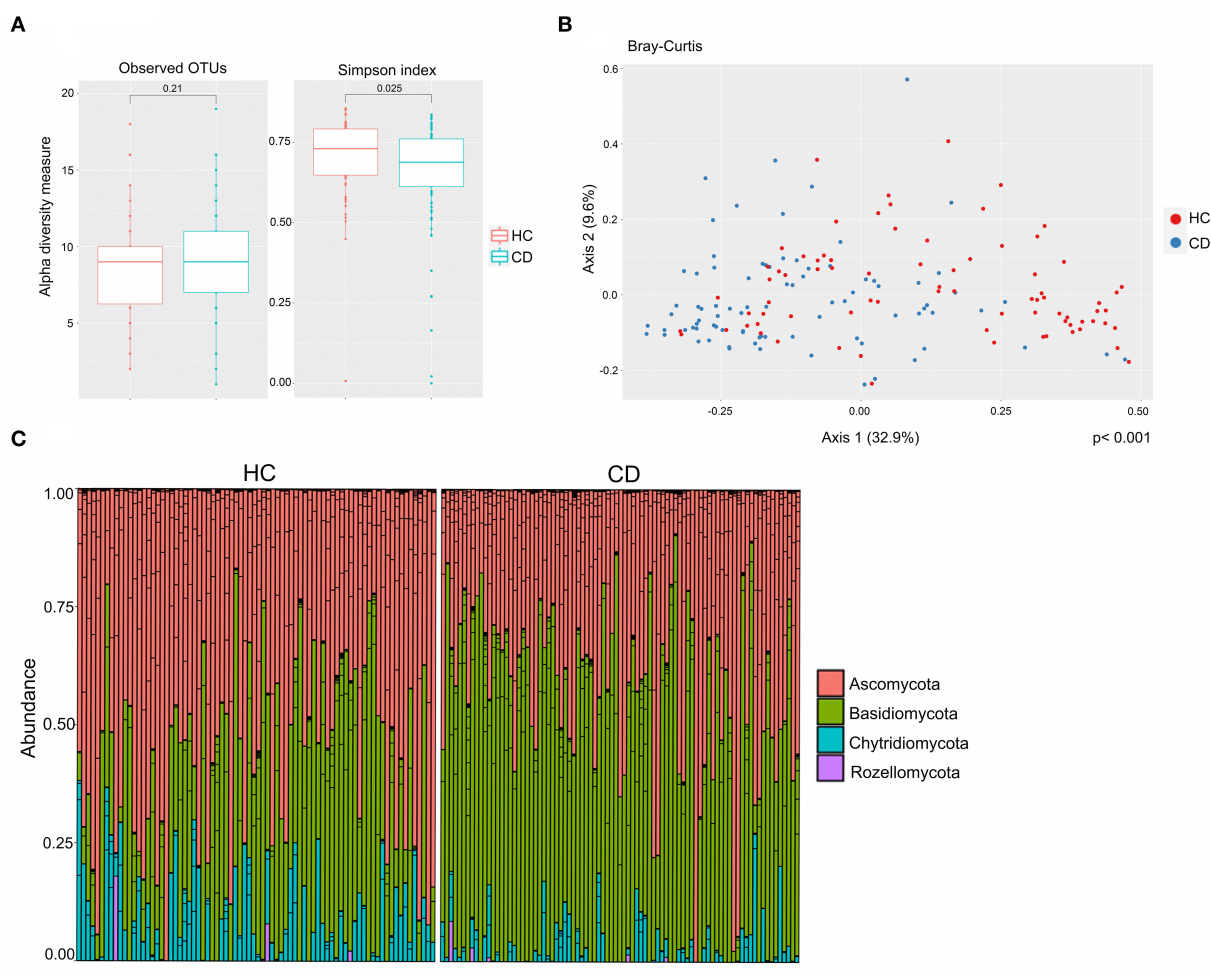
The mucosa-associated mycobiota in Crohn's disease (CD) patients (*n* = 44) was altered in comparison to healthy controls (HC) (*n* = 40). **(A)** Alpha-diversity, according to observed operational taxonomic units (OTUs) (left) and Simpson index (right), boxplots colored according to disease phenotype (HC = red, CD = blue). **(B)** Beta-diversity. Principal coordinates analysis of Bray–Curtis dissimilarity with samples colored according to study group, (CD = blue and HC = red). The fraction of diversity captured by the coordinate is given in percentage on axes 1 and 2. Groups were compared using the Permanova method. **(C)** Relative abundance of fungal phyla in HC and CD patients.

**Figure 2:**
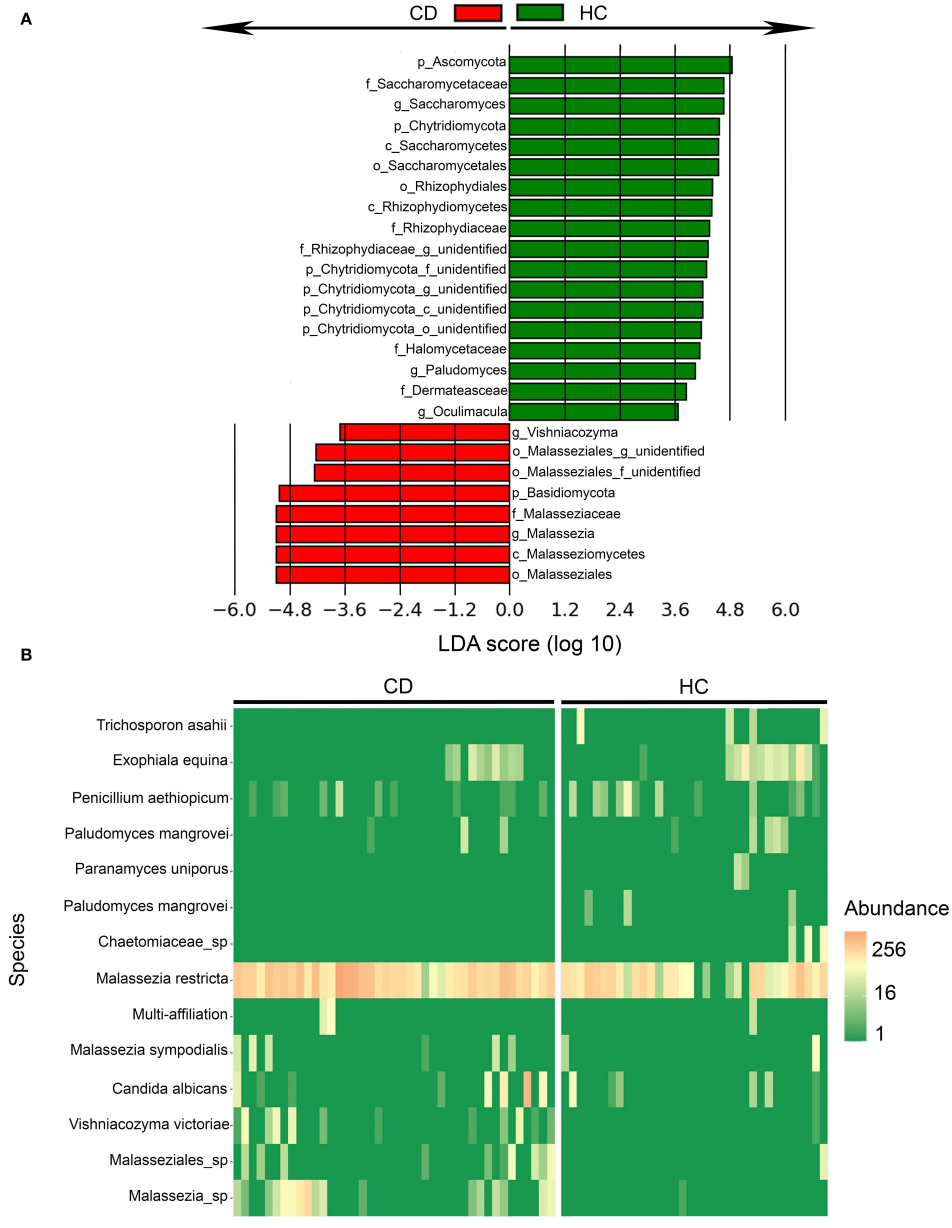
Fungal taxa were differentially abundant in Crohn's disease (CD) patients (*n* = 44) in comparison to healthy controls (HC) (*n* = 40) identified by linear discriminant analysis effect size (LEfSe). **(A)** Fungal taxa overrepresented in CD patients (red) and HC (green) with Linear Discriminant Analysis (LDA) score for differentially abundant fungal taxa. **(B)** Heatmap of differentially abundant fungal species between CD and HC mucosal pinch biopsies sampled 5-cm proximal of the ileocecal valve or ileocolic anastomosis.

CD patients had a lower fungal alpha diversity compared to HC based on the Simpson diversity index (*p* = 0.025), whereas the observed numbers of operational taxonomic units (OTUs) were similar (*p* = 0.21). This implies that the fungal species richness was similar, but that the evenness of fungi was reduced within the CD group compared to HC (Figure 1A). The most prevalent phyla in the samples overall were Ascomycota, Basidiomycota, and Chytridiomycota, and some Rozellomycota were also detected (Figure 1C). In CD patients, the Basidiomycota-to-Ascomycota ratio was increased compared to HC (Supplementary Figure 1A). CD patients also had lower abundances of Chytridiomycota phyla (Supplementary Figure 1A). Beta diversity analysis assessed by Bray–Curtis dissimilarity showed a clustering of the samples according to the disease status (p < 0.001), confirming structural differences in the mycobiota composition between CD patients and HC (Figure 1B). Using LEfSe (39), fungal composition in CD patients and HC were compared and differentially abundant fungi were identified (Figure 2). *Malassezia* and *Vishniacozyma* genera were increased in CD patients, while *Saccharomyces, Paludomyces*, and *Oculimacula* were depleted in comparison to HC (Figure 2A and Supplementary Figure 1B). When the comparison was performed at the species level, CD patients had increased abundances of *Malassezia restricta* as well as *Malassezia sympodialis* and two other *Malassezia* species (Figure 2B). *Candida albicans and Vishniacozyma victoriae* were also increased in CD patients (Figure 2B). In HC, *Trichosporon asahii, Paludomyces mangrovei*, and a species from the Chaetomiaceae family were overrepresented compared to CD patients.

### Mycobiota in the inflamed and proximal non-inflamed ileum in CD patients (*n* = 20)

**Figure 3:**
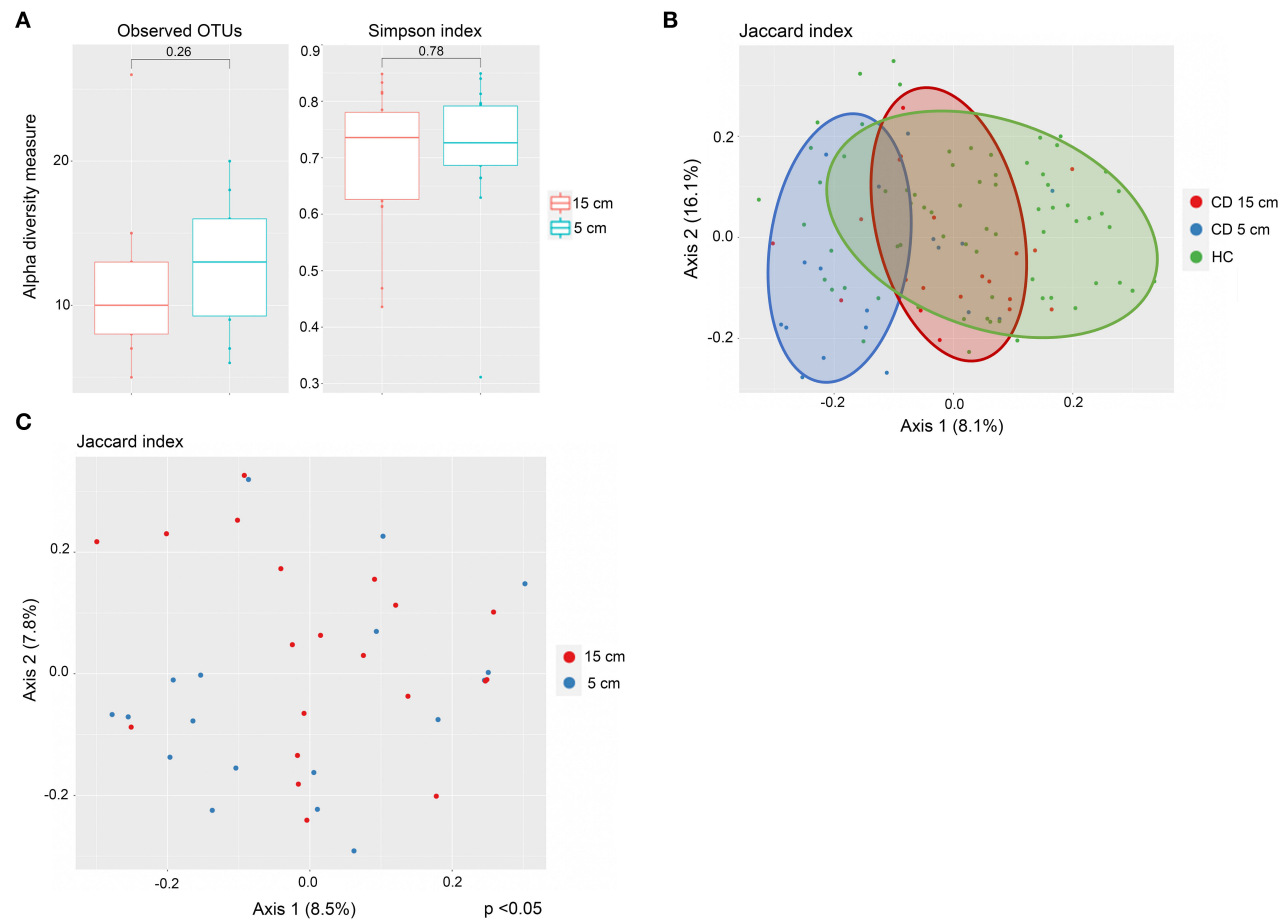
Fungal mycobiota in inflamed and proximally non-inflamed ileal mucosa in Crohn's disease (CD) patients without upper CD involvement (*n* = 20). Biopsies sampled at inflamed 5-cm and non-inflamed 15-cm proximal to the ileocecal valve or ileocolic anastomosis. **(A)** No differences in alpha diversity, according to observed operational taxonomic units (OTUs) (left) and Simpson index (right), boxplots colored according to biopsy location (inflamed 5 cm = blue, non-inflamed 15 cm = red). **(B)** Mycobiota composition in inflamed terminal ileum (blue) and proximally non-inflamed ileum (red) of CD patients (*n* = 20) and in healthy controls (HC) (green) (*n* = 40). Principal coordinates analysis of Jaccard index with samples colored according to disease status (CD and HC) and ileal location. The fraction of diversity captured by the coordinate is given in percentage on axes 1 and 2. **(C)** Different mycobiota composition in inflamed (blue) and proximally non-inflamed ileum (red) according to beta diversity. Principal coordinates analysis of Jaccard index with samples colored according to ileal location. The fraction of diversity captured by the coordinate is given in percentage on axes 1 and 2. Groups were compared using the Permanova method.

**Figure 4:**
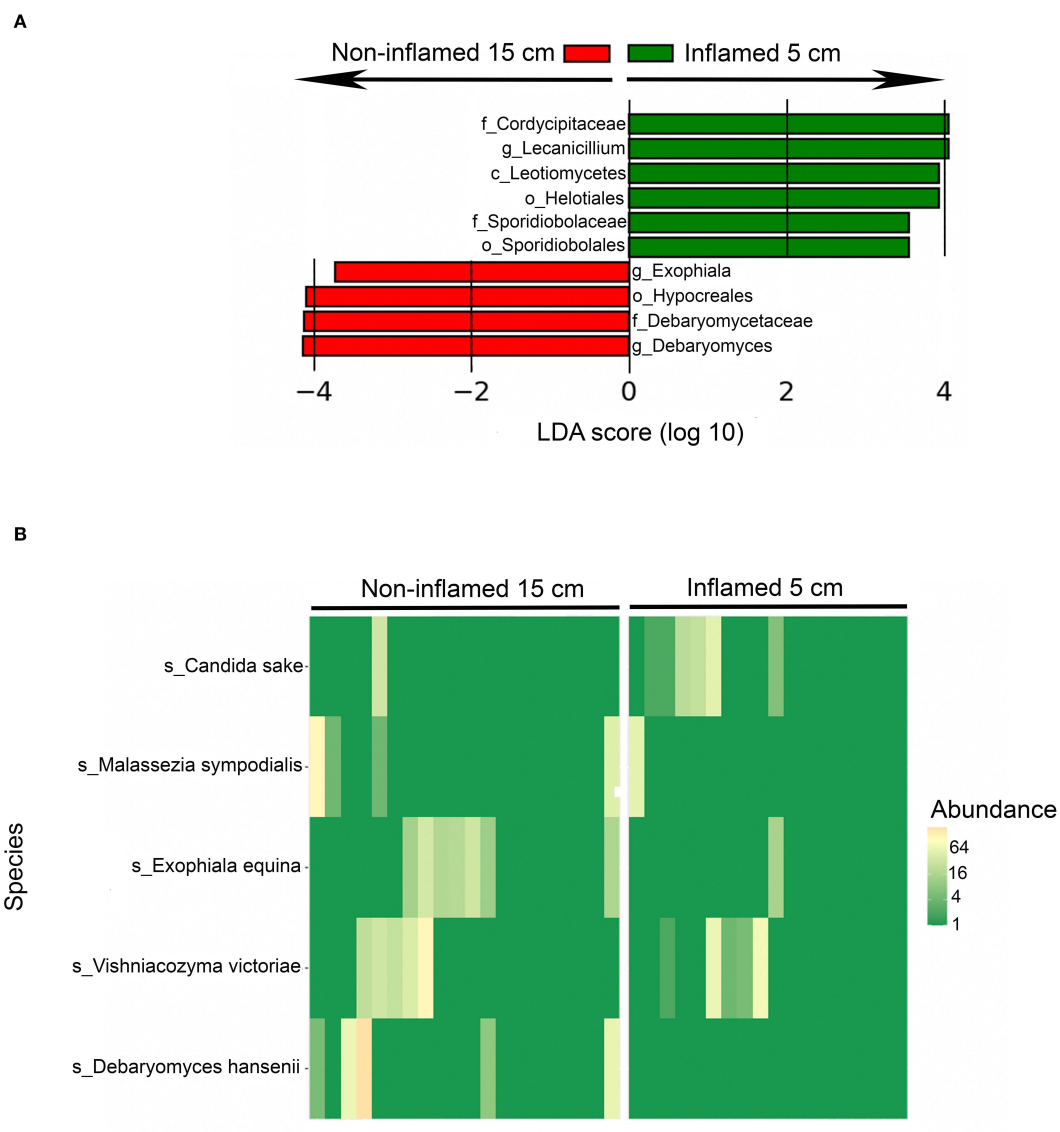
Differentially abundant fungal taxa between inflamed 5-cm vs. proximal non-inflamed 15-cm ileum of Crohn's disease (CD) patients (*n* = 20) with terminal ileitis and no history of upper CD involvement. Biopsies sampled from 5- and 15 cm proximal of the ileocecal valve or ileocolic anastomosis within the same patients. **(A)** Fungal taxa overrepresented in inflamed 5-cm biopsies (green) and non-inflamed 15-cm biopsies (red) in a histogram with Linear Discriminant Analysis (LDA) score computed using linear discriminant analysis effect size (LEfSe). **(B)** Heatmap showing the distribution of differentially abundant fungal species in inflamed 5-cm samples (right) and non-inflamed 15-cm samples (left) identified using LEfSe.

Twenty CD patients had terminal ileitis with an inflamed 5-cm location and a non-inflamed 15-cm location, and no history of upper CD involvement. These patients were analyzed separately. Fungal alpha diversity did not differ between the distal inflamed 5-cm and proximal non-inflamed 15-cm locations in CD patients with terminal ileitis, based on observed OTUs and Simpson index (Figure 3A). Interestingly, on the beta diversity plot assessed by the Jaccard index, which focuses more on low abundant OTUs in comparison to Bray–Curtis dissimilarity, inflamed 5-cm samples clustered furthest away from HC with non-inflamed CD 15-cm samples in an intermediate location (Figure 3B), suggesting a more dysbiotic fungal composition in the distal inflamed ileum. In a beta diversity plot including only CD patients with terminal ileitis, 5- and 15-cm samples were separated clearly (*p* < 0.05) according to the Jaccard index (Figure 3C). When we compared the fungal composition in inflamed 5-cm samples with non-inflamed 15-cm samples using LEfSe, we identified six taxa that were increased at the 5-cm location and four taxa that were increased at the 15-cm location (Figure 4). Cordycipitaceae and Sporidiobolaceae families and *Lecanicillium* genus were overrepresented at the inflamed 5-cm location, whereas *Exophiala* and *Debaryomyces* genera were overrepresented at the non-inflamed 15-cm location. Differentially abundant species were identified using LEfSe are presented in a heatmap (Figure 4B). *Candida sake* was overrepresented at the inflamed 5-cm location. The *Exophiala* and *Debaryomyces* genera, which were increased at the non-inflamed 15-cm location, were identified as *Exophiala equina and Debaryomyces hansenii* (Figure 4B).

### Mycobiota associated with the need for escalation of CD treatment

**Figure 5:**
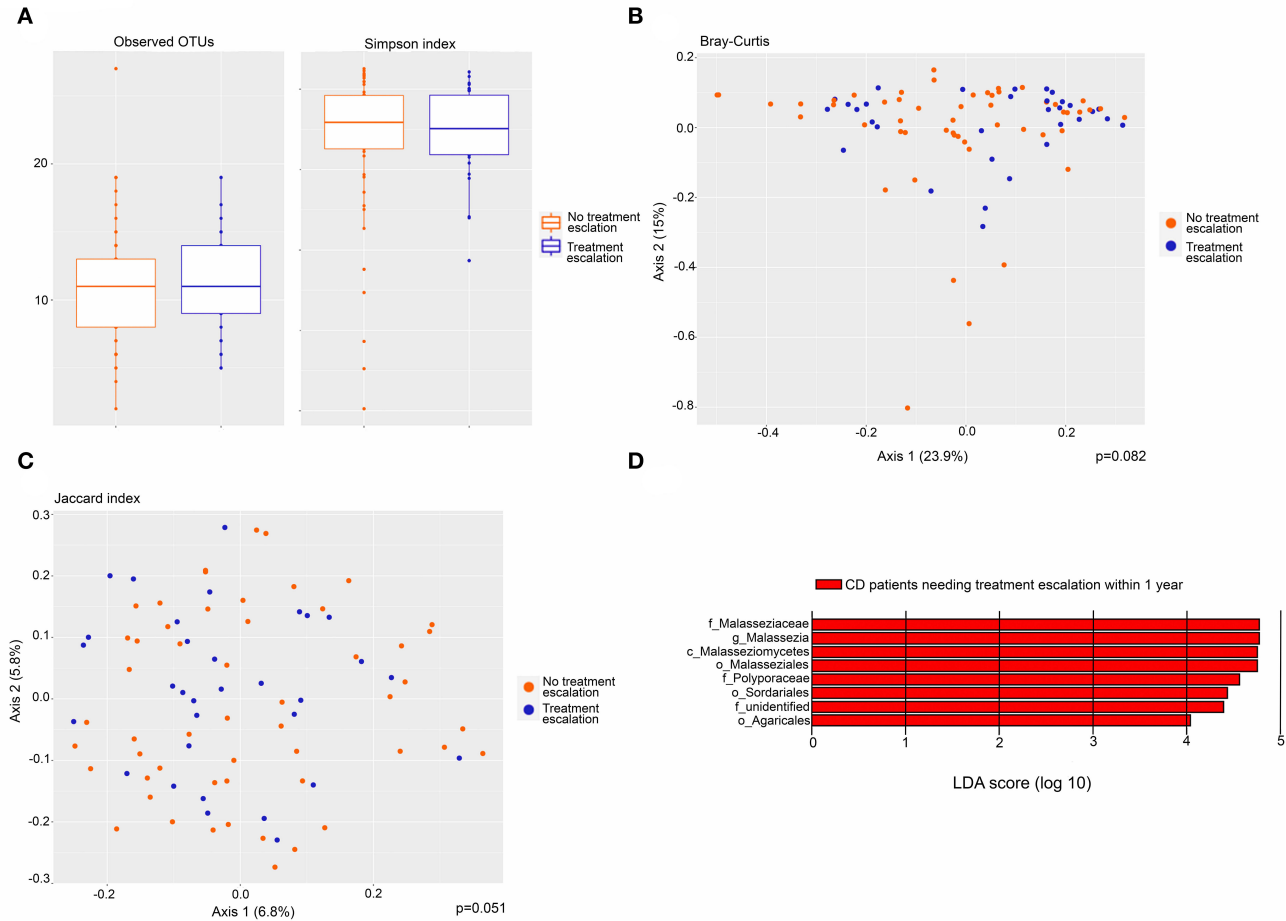
Fungal microbiota in CD patients needing treatment escalation (*n* = 17) within the first year after biopsy sampling compared to CD patients not needing treatment escalation (*n* = 27). **(A)** Fungal alpha-diversity, according to observed operational taxonomic units (OTUs) (left) and Simpson index (right), boxplots colored according to need for treatment escalation (blue) and no need for treatment escalation (red). **(B)** Beta-diversity. Principal coordinates analysis of Bray–Curtis dissimilarity with samples colored according to the need for treatment escalation (blue) and no need for treatment escalation (red). The fraction of diversity captured by the coordinate is given in percentage on axes 1 and 2. Groups were compared using the Permanova method. **(C)** Principal coordinates analysis of Jaccard index with samples colored according to need for treatment escalation (yes = blue, no = red). The fraction of diversity captured by the coordinate is given in percentage on axes 1 and 2. Groups were compared using the Permanova method. **(D)** Fungal taxa overrepresented in CD patients needing treatment escalation within 1 year compared to CD patients not needing treatment escalation, illustrated in a histogram with Linear Discriminant Analysis (LDA) score computed using linear discriminant analysis effect size (LEfSe).

CD patients were stratified by their need for treatment escalation within the first year after biopsy collection and the mycobiota was compared across this variable. Seventeen of 44 patients received additional anti-inflammatory treatment. Of those 17 patients, 15 received escalation of anti-inflammatory medical treatment, while two patients underwent surgery (Supplementary Table 1). There was no difference in alpha diversity based on observed OTUs and Simpson index (Figure 5A). Beta diversity assessed by Bray–Curtis dissimilarity (*p* =0.082) and Jaccard index (*p* =0.051) did not differ significantly between CD patients with and without the need for treatment escalation (Figures 5B,C). In a differential analysis using LefSE, we found fungi at several taxonomic levels to be more abundant in CD patients needing treatment escalation within the first year after sampling (Figure 5D). These fungal taxa can possibly be predictive of poor prognosis in CD patients. In particular, the Malasseziaceae family and *Malassezia* genus were more abundant in CD patients needing treatment escalation.

### Effect of anti-TNF treatment on mycobiota

**Figure 6:**
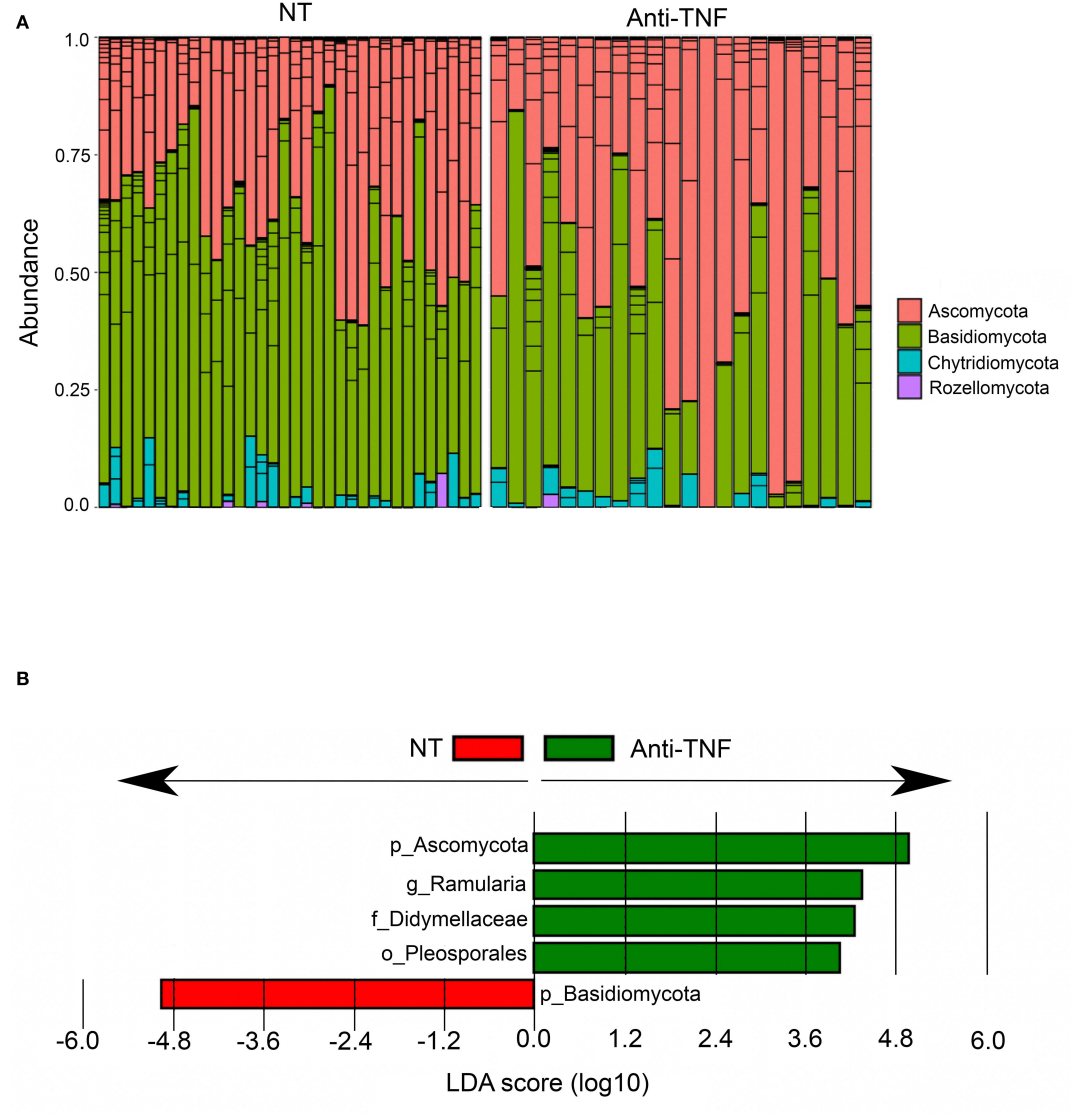
Differentially abundant fungal taxa between Crohn's disease (CD) patients currently using anti-TNF agents (Anti-TNF) (*n* = 11) compared to CD patients with no medical treatment (NT) (*n* = 18). **(A)** Relative abundance of fungal phyla in CD patients with no medical treatment (NT) and anti-TNF treated CD patients. **(B)** Fungal taxa overrepresented in anti-TNF-treated CD patients (green) and in CD patients with no medical treatment (NT) (red) illustrated in a histogram with Linear Discriminant Analysis (LDA) score computed using linear discriminant analysis effect size (LEfSe).

Eleven patients under current treatment with anti-TNF agents were compared to 18 patients with no current medical treatment for CD (Table 2). We found no differences in alpha- or beta diversities between these groups (Supplementary Figure 2). Interestingly, differential analysis with LefSE identified anti-TNF users to have higher abundances of Ascomycota and correspondingly lower abundances of Basidiomycota (Figure 6A), whereas in CD patients with no current treatment, the abundances of Basidiomycota (Figures 6A,B) was high. Correspondingly, the whole CD cohort had increased levels of Basidiomycota and reduced levels of Ascomycota compared to HC, as described earlier. When comparing anti-TNF naïve patients (never-users), *n* = 23, with ever-users (historically) of anti-TNF treatment, *n* = 21 (Table 2), we found a similar mycobiota composition according to both alpha diversity and beta diversity (Supplementary Figures 3A–C).

### Mycobiota according to ileal inflammation and sub-location in CD patients overall

Within the whole CD cohort (*n* = 44), inflammation (both endoscopic and histologic) was not associated with an altered mycobiota diversity or composition, based on observed OTUs, Simpson index, and Bray–Curtis dissimilarity (Supplementary Figure 4). This argues that endoscopic and histologic inflammation *per se* does not dominantly alter the fungal microbiota. Similarly, ileal location (5 vs. 15 cm) was not associated with an altered fungal microbiota diversity or composition in the CD cohort (*n* = 44) (Supplementary Figure 5). These findings imply that the altered fungal microbiota in CD terminal ileitis could not be explained by the effect of location or inflammation alone and is in accordance with our previous findings suggesting that mucosa-associated bacterial alterations in CD are also present across locations and independent of inflammation (24).

## Discussion

This study investigated the mucosa-associated fungal microbiota in CD and compared the inflamed and proximal non-inflamed ileum within CD patients. Previous reports have found altered fungal composition in CD compared to HC (14, 16, 22, 23, 40). We found that the mucosa-associated mycobiota in the ileum of CD patients had reduced alpha diversity based on the Simpson index, but a similar number of observed OTUs compared to HC, implicating similar species richness, but reduced evenness in CD patients. CD patients had an increased Basidiomycota-to-Ascomycota ratio as reported by others (14, 40), but also an altered mycobiota composition characterized by a significant gain of *Malassezia* and loss of *Saccharomyces*. At species level, *Malassezia* was identified as *Malassezia restricta* and *Malassezia sympodialis*. The expansion of *Malassezia restricta* in CD has been reported previously (14, 41), particularly in CD patients carrying the *CARD9* risk allele (14). Depletion of *Saccharomyces* has been described in feces from IBD patients, where *Saccharomyces* was positively correlated with abundances of bacteria depleted in IBD, such as the butyrate-producing *Roseburia, Blautia*, and *Ruminococcus* genera (40, 42). Several *Saccharomyces* spp. have been suggested to have anti-inflammatory effects (40, 43–45).

We found increased abundance of *C. albicans* in the ileal mucosa of CD patients. *C. albicans* has been proposed to promote IBD by increasing the inflammatory response, and due to its increased abundance during inflammation, a vicious circle is created (45, 46). The fecal abundance of *Candida* before fecal microbiota transplantation (FMT) in UC patients has been associated with therapeutic response, and effects of FMT may be mediated by a reduction in *Candida* abundance (47). Increased abundances of *Candida albicans* and *Candida glabrata* have previously been found in fecal and colonic samples, respectively, from CD patients (16, 40, 48). To the best of our knowledge, this is the first study to confirm increased *Candida* in the ileal mucosa, thus supporting a clinical relevance. Interestingly, increased abundances of *Candida tropicalis* in fecal samples from CD patients have been positively correlated to ASCA concentrations (49). *Candida* is also extensively involved in bacterial interactions, demonstrating a significant influence on microbiome composition (45, 49). However, the reports show opposite effects depending on the experimental setup. Studies following the bacterial community reassembly after antibiotic treatment showed the influence of *C. albicans* on the bacterial diversity levels and possible influence on *Lachnospiraceae* colonization, a family with recognized positive effects on gut health (50, 51). In a mucosal model evaluating mouth and gut colonization, *C. albicans* triggered a dysbiosis characterized by a bloom of *Enterococcus* strains associated with increased epithelial permeability and susceptibility to invasive infections (52).

The terminal ileum is the predilection site for primary and recurrent CD and we, therefore, specifically analyzed the mucosa-associated mycobiota in the inflamed and proximal non-inflamed mucosa in patients with terminal ileitis. The alpha diversity in inflamed and proximal non-inflamed mucosa did not differ; however, a separation on beta diversity plots suggested an altered and more dysbiotic fungal composition in the inflamed ileum compared to proximal non-inflamed ileum and healthy mucosa of controls. *Lecanicillium* genera and *Candida sake* sp. were increased in the inflamed mucosa, whereas *Exophiala equina and Debaryomyces hansenii* were increased in the proximal non-inflamed mucosa. *C. sake* is frequently found in the feces of healthy humans (31), it can cause rare invasive candidemia (53) but have, however, not been associated with IBD to our knowledge. Indeed, *C. sake* is used as a biocontrol agent in the food industry to limit the decay of apples due to mold (54, 55). The literature on *E. equina* is scarce, but *E. equina* has been identified in subcutaneous abscesses with histologically granulomatous inflammation (56) and *Exophiala* has been associated with primary sclerosing cholangitis (57). *D. hansenii* is a commensal gut fungus that is found in feces of healthy adults and reported to be increased in feces of infants (31, 58), it is also frequently found in foods such as meat, fruit, cheese, beer, and wine (59). Jain et al. have recently reported *D. hansenii* to be enriched and completely dominant in inflamed compared to the non-inflamed ileum in 16 CD patients from two different cohorts (23), which is the opposite of our and Liguori et al. (16) findings. Jain et al. did not consider the relative location of inflamed and non-inflamed samples, and slight differences in DNA isolation protocols between studies could also affect results. However, *D. hansenii* was found to impair tissue healing in mice models, but intestinal damage was required for *D. hansenii* to have detrimental effect (23). The mentioned differences between studies could hypothetically be explained by the transfer of *D. hansenii* from the proximal non-inflamed mucosa to the ileum downstream and reduce wound healing, consistent with early descriptions of a beneficial effect of fecal stream diversion (2, 3).

Interestingly, we found that increased abundance of *Malassezia* genus with corresponding increases of Malasseziaceae at the family level, Malasseziomycetes class, and Malasseziales order were associated with the need for treatment escalation within 1-year follow-up. The association suggests that *Malassezia* does not only characterize CD in our and other patient cohorts (14) but it may also affect the disease course and represent a poor prognostic factor. Larger prospective studies to examine this observation would be of great interest.

Anti-TNF treatment was also associated with alterations of the fungal microbiota. CD patients on current anti-TNF treatment had lower abundances of Basidiomycota compared to CD patients not receiving medical treatment. CD patients in general have an increased Basidiomycota-to-Ascomycota ratio compared to HC, as reported in this cohort as well as by others (14, 40). A recent study investigating the bacterial and fungal communities in fecal samples before and after initiation of anti-TNF treatment found that both fungal and bacterial microbiota composition differed between anti-TNF-responders and non-responders (60), arguing that the microbiome composition is relevant for pharmacological therapy. Anti-TNF treatment could modulate the mycobiota in a potentially beneficial direction since it reversed the fungal community toward a lower Basidiomycota-to-Ascomycota ratio which is found in healthy subjects. However, the finding should be verified in larger cohorts, and the mechanisms by which this occurs need to be evaluated.

Neither endoscopic nor histologic inflammation was associated with an increased number of OTUs or altered mycobiota according to other alpha- or beta-diversity measures. On the contrary, fungal richness and diversity have previously been found to be increased in inflamed vs. non-inflamed mucosa assessed by PCR and Denaturing Gel Gradient Electrophoresis; however, the method is less sensitive in terms of taxa identification and diversity measures compared to ITS-sequencing (61). We found that ileal sub-location seems to neither impact mycobiota diversity nor its composition. Finally, patients using anti-TNF agents did not have a mycobiome that differed from CD patients not receiving any anti-inflammatory treatment. This observation strengthens the hypothesis that altered ileal mycobiome in CD patients is related to the disease *per se*.

The role of fungi in IBD has been described and acknowledged (62), consequently the fungal microbiome is a potential therapeutic target. Factors known to affect the mycobiome include diet, antibacterial and antifungal agents, and gut bacteria (9, 45, 49, 63, 64). The risk of CD was associated with cumulative antibiotic exposure in a Swedish national cohort (65). Antibacterial therapy increases fungal abundances in fecal samples (64, 66), suggesting that fungi could mediate the increased risk of CD after exposure to antibacterial agents (46). Ingestion of meat, eggs, and cheeses seems to increase the fecal fungal load compared to vegetarian food (67), and correspondingly, fiber and fruit reduce the risk of CD in epidemiological studies (68). It has recently been found that a proportion of secretory IgA (sIgA), which have an important gut barrier function, is induced by and directed toward intestinal fungi (69). The production of sIgAs that target and coat certain fungi may be dysregulated in CD. The previously recognized risk factors and prognostic factors for IBD could be mediated by alterations of intestinal fungi. Food that either contains fungi or otherwise alters the intestinal composition of fungi could be of importance (63, 67), but considering the complex interactions between bacteria and fungi, drugs that alter microbial composition including anti-bacterial agents and proton-pump inhibitors could also be implicated (70). Before establishing treatment strategies aiming to maintain or restore a health-promoting mycobiome, prospective and interventional studies with careful monitoring of intestinal fungi are needed. Given the widespread interest in microbiota research, it would also be valuable if the numerous studies of fecal microbiota transplantation also included sequencing of not only bacteria but also fungi and viruses. Oral anti-fungal agents have the potential to reduce inflammation in IBD, and this approach should be explored further (71).

There are several challenges within the field of mycobiota research. Several methodological steps harbor a potential for variation which can impact reported mycobiota composition. This includes the method of sample collection which varies from endoscopic biopsies (22), endoscopic water-lavage samples (14), surgical samples (23), or even a combination (16). Furthermore, the storage of samples, DNA isolation protocol, choice of primer (ITS1 or ITS2), sequencing protocol, and bioinformatic pipeline vary.

Strengths of the study include analysis of the mucosa-associated microbiota, which represent only a section of the entire gut microbiota, but due to its location interacts with the host cells and the immune system, which provides original data compared to the most common studies on fecal composition. Additionally, the mucosa-associated mycobiota was assessed in the highest number of CD patients to date (14, 16, 22, 23), and the sequencing analysis of high quality and the majority of sequences were taxonomically classified. Furthermore, the clinical course was followed for 1 year after biopsy collection. Limitations include the observational study design, heterogeneity of the CD duration, and previous and current medical and surgical treatment that may affect the mycobiota. We have not correlated the bacterial and fungal microbiota, and interactions between bacteria and fungi occurring have not been assessed.

In conclusion, this relatively large study describes the mucosa-associated mycobiota in the inflamed and proximally non-inflamed ileum in CD patients and confirms several alterations found in other cohorts. We have also identified fungal taxa which are associated with the need for treatment escalation in CD. The mycobiota composition in the inflamed ileum and proximal non-inflamed ileum differ and may play a role in CD pathogenesis.

## Data availability statement

The sequencing data underlying this article are available at NCBI with accession number PRJNA850908.

## Ethics statement

The studies involving human participants were reviewed and approved by Regional Committee for Medical and Health Research Ethics, Central Norway (approval reference, 2016/2164). The patients/participants provided their written informed consent to participate in this study.

## Author contributions

MO, TM, and RF were responsible for the study design. MO and RF were responsible for data acquisition. MO, MR, OR, VB, AG, and ER were responsible for analyses of biological material and data analysis. MO, MR, and RF interpreted the results and drafted the manuscript. MO, MR, VB, AG, ER, TM, AS, HS, and RF contributed to the critical revision of the manuscript. All authors contributed to the article and approved the submitted version.

## Funding

This study was funded by the Liaison Committee between Central Norway Regional Health Authority (RHA), the Norwegian University of Science and Technology (NTNU) (2016/29014), and by St. Olav's Hospital, Trondheim University Hospital. The GCF is funded by the Faculty of Medicine and Health Sciences at NTNU and Central Norway Regional Health Authority.

## Conflict of interest

The authors declare that the research was conducted in the absence of any commercial or financial relationships that could be construed as a potential conflict of interest.

## Publisher's note

All claims expressed in this article are solely those of the authors and do not necessarily represent those of their affiliated organizations, or those of the publisher, the editors and the reviewers. Any product that may be evaluated in this article, or claim that may be made by its manufacturer, is not guaranteed or endorsed by the publisher.

## References

[1] Torres J Mehandru S Colombel JF Peyrin-Biroulet L. Crohn's Disease. Lancet. (2017) 389:1741–55. 10.1016/S0140-6736(16)31711-1 27914655

[2] Rutgeerts P Goboes K Peeters M Hiele M Penninckx F Aerts R. Effect of faecal stream diversion on recurrence of crohn's disease in the neoterminal ileum. Lancet. (1991) 338:771–4. 10.1016/0140-6736(91)90663-A 1681159

[3] D'Haens GR Geboes K Peeters M Baert F Penninckx F Rutgeerts P. Early lesions of recurrent crohn's disease caused by infusion of intestinal contents in excluded ileum. Gastroenterology. (1998) 114:262–7. 10.1016/S0016-5085(98)70476-7 9453485

[4] Fornaro R Caratto E Caratto M Fornaro F Caristo G Frascio M. Post-operative recurrence in crohn's disease. Critical analysis of potential risk factors: an update. Surgeon. (2015) 13:330–47. 10.1016/j.surge.2015.04.002 26049657

[5] Rutgeerts P Geboes K Vantrappen G Kerremans R Coenegrachts JL Coremans G. Natural history of recurrent crohn's disease at the ileocolonic anastomosis after curative surgery. Gut. (1984) 25:665–72. 10.1136/gut.25.6.665 6735250 PMC1432363

[6] Sokol H Brot L Stefanescu C Auzolle C Barnich N Buisson A. Prominence of ileal mucosa-associated microbiota to predict postoperative endoscopic recurrence in crohn's disease. Gut. (2019) 69:462–72. 10.1136/gutjnl-2019-318719 31142586

[7] Yilmaz B Juillerat P Oyas O Ramon C Bravo FD Franc Y. Microbial network disturbances in relapsing refractory crohn's disease. Nat Med. (2019) 25:323–36. 10.1038/s41591-018-0308-z 30664783

[8] Sokol H Pigneur B Watterlot L Lakhdari O Bermudez-Humaran LG Gratadoux JJ. Faecalibacterium prausnitzii is an anti-inflammatory commensal bacterium identified by gut microbiota analysis of crohn disease patients. Proc Natl Acad Sci U S A. (2008) 105:16731–6. 10.1073/pnas.0804812105 18936492 PMC2575488

[9] Mukherjee PK Sendid B Hoarau G Colombel JF Poulain D Ghannoum MA. Mycobiota in gastrointestinal diseases. Nat Rev Gastroenterol Hepatol. (2015) 12:77–87. 10.1038/nrgastro.2014.188 25385227

[10] Main J McKenzie H Yeaman GR Kerr MA Robson D Pennington CR. Antibody to saccharomyces cerevisiae (bakers' yeast) in Crohn's disease. Bmj. (1988) 297:1105–6. 10.1136/bmj.297.6656.1105 3143445 PMC1834894

[11] Jostins L Ripke S Weersma RK Duerr RH McGovern DP Hui KY. Host-microbe interactions have shaped the genetic architecture of inflammatory bowel disease. Nature. (2012) 491:119–24. 10.1038/nature11582 23128233 PMC3491803

[12] Rivas MA Beaudoin M Gardet A Stevens C Sharma Y Zhang CK. Deep resequencing of gwas loci identifies independent rare variants associated with inflammatory bowel disease. Nat Genet. (2011) 43:1066–73. 10.1038/ng.952 21983784 PMC3378381

[13] Glocker EO Hennigs A Nabavi M Schäffer AA Woellner C Salzer U. A homozygous card9 mutation in a family with susceptibility to fungal infections. N Engl J Med. (2009) 361:1727–35. 10.1056/NEJMoa0810719 19864672 PMC2793117

[14] Limon JJ Tang J Li D Wolf AJ Michelsen KS Funari V. Malassezia is associated with crohn's disease and exacerbates colitis in mouse models. Cell Host Microbe. (2019) 25:377–88.e6. 10.1016/j.chom.2019.01.007 30850233 PMC6417942

[15] Underhill D Braun J. Current understanding of fungal microflora in inflammatory bowel disease pathogenesis. Inflamm Bowel Dis. (2008) 14:1147–53. 10.1002/ibd.20402 18286647 PMC3752988

[16] Liguori G Lamas B Richard ML Brandi G da Costa G Hoffmann TW. Fungal dysbiosis in mucosa-associated microbiota of crohn's disease patients. J Crohns Colitis. (2016) 10:296–305. 10.1093/ecco-jcc/jjv209 26574491 PMC4957473

[17] Ott SJ Kuhbacher T Musfeldt M Rosenstiel P Hellmig S Rehman A. Fungi and inflammatory bowel diseases: alterations of composition and diversity. Scand J Gastroenterol. (2008) 43:831–41. 10.1080/00365520801935434 18584522

[18] Zmora N Zilberman-Schapira G Suez J Mor U Dori-Bachash M Bashiardes S. Personalized gut mucosal colonization resistance to empiric probiotics is associated with unique host and microbiome features. Cell. (2018) 174:1388–405.e21. 10.1016/j.cell.2018.08.041 30193112

[19] Gevers D Kugathasan S Denson LA Vazquez-Baeza Y Van Treuren W Ren B. The treatment-naive microbiome in new-onset crohn's disease. Cell Host Microbe. (2014) 15:382–92. 10.1016/j.chom.2014.02.005 24629344 PMC4059512

[20] Morgan XC Tickle TL Sokol H Gevers D Devaney KL Ward DV. Dysfunction of the intestinal microbiome in inflammatory bowel disease and treatment. Genome Biol. (2012) 13:R79. 10.1186/gb-2012-13-9-r79 23013615 PMC3506950

[21] Pittayanon R Lau JT Leontiadis GI Tse F Yuan Y Surette M. Differences in gut microbiota in patients with vs without inflammatory bowel diseases: a systematic review. Gastroenterology. (2020) 158:930–46.e1. 10.1053/j.gastro.2019.11.294 31812509

[22] El Mouzan M Wang F Al Mofarreh M Menon R Al Barrag A Korolev KS. Fungal microbiota profile in newly diagnosed treatment-naive children with crohn's disease. J Crohns Colitis. (2017) 11:586–92. 10.1093/ecco-jcc/jjw197 27811291

[23] Jain U Ver Heul AM Xiong S Gregory MH Demers EG Kern JT. Debaryomyces is enriched in crohn's disease intestinal tissue and impairs healing in mice. Science. (2021) 371:1154–9. 10.1126/science.abd0919 33707263 PMC10114606

[24] Olaisen M Flatberg A Granlund AVB Røyset ES Martinsen TC Sandvik AK. Bacterial mucosa-associated microbiome in inflamed and proximal noninflamed ileum of patients with crohn's disease. Inflamm Bowel Dis. (2020) 27:12–24. 10.1093/ibd/izaa107 32448900 PMC7737161

[25] Silverberg MS Satsangi J Ahmad T Arnott ID Bernstein CN Brant SR. Toward an integrated clinical, molecular and serological classification of inflammatory bowel disease: report of a working party of the 2005 montreal world congress of gastroenterology. Can J Gastroenterol. (2005) 19:5a−36a. 10.1155/2005/269076 16151544

[26] Mearin F Lacy BE Chang L Chey WD Lembo AJ Simren M. Bowel disorders. Gastroenterology. (2016) 150:1393–407. 10.1053/j.gastro.2016.02.031 27144627

[27] Rutgeerts P Geboes K Vantrappen G Beyls J Kerremans R Hiele M. Predictability of the postoperative course of crohn's disease. Gastroenterology. (1990) 99:956–63. 10.1016/0016-5085(90)90613-6 2394349

[28] Mosli MH Feagan BG Zou G Sandborn WJ D'Haens G Khanna R. Development and validation of a histological index for Uc. Gut. (2017) 66:50–8. 10.1136/gutjnl-2015-310393 26475633

[29] Novak G Parker CE Pai RK MacDonald JK Feagan BG Sandborn WJ. Histologic scoring indices for evaluation of disease activity in crohn's disease. Cochrane Database Syst Rev. (2017) 7:Cd012351. 10.1002/14651858.CD012351.pub2 28731502 PMC6483549

[30] Sandborn WJ Feagan BG Hanauer SB Lochs H Lofberg R Modigliani R. A review of activity indices and efficacy endpoints for clinical trials of medical therapy in adults with crohn's disease. Gastroenterology. (2002) 122:512–30. 10.1053/gast.2002.31072 11832465

[31] Nash AK Auchtung TA Wong MC Smith DP Gesell JR Ross MC. The gut mycobiome of the human microbiome project healthy cohort. Microbiome. (2017) 5:153. 10.1186/s40168-017-0373-4 29178920 PMC5702186

[32] Gow NAR Latge JP Munro CA. The fungal cell wall: structure, biosynthesis, and function. Microbiol Spectr. (2017) 5:5. 10.1128/microbiolspec.FUNK-0035-2016 28513415 PMC11687499

[33] Tang J Iliev ID Brown J Underhill DM Funari VA. Mycobiome: approaches to analysis of intestinal fungi. J Immunol Methods. (2015) 421:112–21. 10.1016/j.jim.2015.04.004 25891793 PMC4451377

[34] Illumina. Fungal Metagenomic Sequencing Demonstrated Protocol (2019). Available online at: https://support.illumina.com/content/dam/illumina-support/documents/documentation/chemistry_documentation/metagenomic/fungal-metagenomic-demonstrated-protocol-1000000064940-01.pdf (accessed September 17, 2022).

[35] Frogs Pipeline. Available online at: http://frogs.toulouse.inra.fr (accessed September 17, 2022).

[36] Escudié F Auer L Bernard M Mariadassou M Cauquil L Vidal K. Frogs: find, rapidly, otus with galaxy solution. Bioinformatics. (2018) 34:1287–94. 10.1093/bioinformatics/btx791 29228191

[37] Nilsson RH Larsson KH Taylor AFS Bengtsson-Palme J Jeppesen TS Schigel D. The unite database for molecular identification of fungi: handling dark taxa and parallel taxonomic classifications. Nucleic Acids Res. (2019) 47:D259–d64. 10.1093/nar/gky1022 30371820 PMC6324048

[38] Love MI Huber W Anders S. Moderated estimation of fold change and dispersion for rna-seq data with deseq2. Genome Biol. (2014) 15:550. 10.1186/s13059-014-0550-8 25516281 PMC4302049

[39] Segata N Izard J Waldron L Gevers D Miropolsky L Garrett WS. Metagenomic biomarker discovery and explanation. Genome Biol. (2011) 12:R60. 10.1186/gb-2011-12-6-r60 21702898 PMC3218848

[40] Sokol H Leducq V Aschard H Pham HP Jegou S Landman C. Fungal microbiota dysbiosis in ibd. Gut. (2017) 66:1039–48. 10.1136/gutjnl-2015-310746 26843508 PMC5532459

[41] Ha CWY Martin A Sepich-Poore GD Shi B Wang Y Gouin K. Translocation of viable gut microbiota to mesenteric adipose drives formation of creeping fat in humans. Cell. (2020) 183:666–83.e17. 10.1016/j.cell.2020.09.009 32991841 PMC7521382

[42] Takahashi K Nishida A Fujimoto T Fujii M Shioya M Imaeda H. Reduced abundance of butyrate-producing bacteria species in the fecal microbial community in crohn's disease. Digestion. (2016) 93:59–65. 10.1159/000441768 26789999

[43] McIlroy J Ianiro G Mukhopadhya I Hansen R Hold GL. Review article: the gut microbiome in inflammatory bowel disease-avenues for microbial management. Aliment Pharmacol Ther. (2018) 47:26–42. 10.1111/apt.14384 29034981

[44] Guslandi M Mezzi G Sorghi M Testoni PA. Saccharomyces boulardii in maintenance treatment of crohn's disease. Dig Dis Sci. (2000) 45:1462–4. 10.1023/A:1005588911207 10961730

[45] Richard ML Sokol H. The gut mycobiota: insights into analysis, environmental interactions and role in gastrointestinal diseases. Nat Rev Gastroenterol Hepatol. (2019) 16:331–45. 10.1038/s41575-019-0121-2 30824884

[46] Sokol H. Antibiotics: a trigger for inflammatory bowel disease? Lancet Gastroenterol Hepatol. (2020) 5:956–7. 10.1016/S2468-1253(20)30208-9 32818436

[47] Leonardi I Paramsothy S Doron I Semon A Kaakoush NO Clemente JC. Fungal trans-kingdom dynamics linked to responsiveness to fecal microbiota transplantation (fmt) therapy in ulcerative colitis. Cell Host Microbe. (2020) 27:823–9.e3. 10.1016/j.chom.2020.03.006 32298656 PMC8647676

[48] Standaert-Vitse A Sendid B Joossens M François N Vandewalle-El Khoury P Branche J. Candida albicans colonization and asca in familial crohn's disease. Am J Gastroenterol. (2009) 104:1745–53. 10.1038/ajg.2009.225 19471251

[49] Hoarau G Mukherjee PK Gower-Rousseau C Hager C Chandra J Retuerto MA. Bacteriome and mycobiome interactions underscore microbial dysbiosis in familial crohn's disease. mBio. (2016) 7:e01250–16. 10.1128/mBio.01250-16 27651359 PMC5030358

[50] Mason KL Erb Downward JR Mason KD Falkowski NR Eaton KA Kao JY. Candida albicans and bacterial microbiota interactions in the cecum during recolonization following broad-spectrum antibiotic therapy. Infect Immun. (2012) 80:3371–80. 10.1128/IAI.00449-12 22778094 PMC3457555

[51] Erb Downward JR Falkowski NR Mason KL Muraglia R Huffnagle GB. Modulation of post-antibiotic bacterial community reassembly and host response by candida albicans. Sci Rep. (2013) 3:2191. 10.1038/srep02191 23846617 PMC3709164

[52] Bertolini M Ranjan A Thompson A Diaz PI Sobue T Maas K. Candida albicans induces mucosal bacterial dysbiosis that promotes invasive infection. PLoS Pathog. (2019) 15:e1007717. 10.1371/journal.ppat.1007717 31009520 PMC6497318

[53] Juneja D Borah AK Nasa P Singh O Javeri Y Dang R. Candida sake candidaemia in non-neutropenic critically ill patients: a case series. Crit Care Resusc. (2011) 13:187–91. 21880007

[54] Nunes C Usall J Teixidó N Viñas I. Improvement of candida sake biocontrol activity against post-harvest decay by the addition of ammonium molybdate. J Appl Microbiol. (2002) 92:927–35. 10.1046/j.1365-2672.2002.01602.x 11972698

[55] Morales H Sanchis V Usall J Ramos AJ Marín S. Effect of biocontrol agents candida sake and pantoea agglomerans on penicillium expansum growth and patulin accumulation in apples. Int J Food Microbiol. (2008) 122:61–7. 10.1016/j.ijfoodmicro.2007.11.056 18191492

[56] Najafzadeh MJ Suh MK Lee MH Ha GY Kim JR Kim TH. Subcutaneous phaeohyphomycosis caused by exophiala equina, with susceptibility to eight antifungal drugs. J Med Microbiol. (2013) 62:797–800. 10.1099/jmm.0.057406-0 23449873

[57] Lemoinne S Kemgang A Ben Belkacem K Straube M Jegou S Corpechot C. Fungi participate in the dysbiosis of gut microbiota in patients with primary sclerosing cholangitis. Gut. (2020) 69:92–102. 10.1136/gutjnl-2018-317791 31003979

[58] Schei K Avershina E Øien T Rudi K Follestad T Salamati S. Early gut mycobiota and mother-offspring transfer. Microbiome. (2017) 5:107. 10.1186/s40168-017-0319-x 28837002 PMC5571498

[59] Breuer U Harms H. Debaryomyces hansenii–an extremophilic yeast with biotechnological potential. Yeast. (2006) 23:415–37. 10.1002/yea.1374 16652409

[60] Ventin-Holmberg R Eberl A Saqib S Korpela K Virtanen S Sipponen T. Bacterial and fungal profiles as markers of infliximab drug response in inflammatory bowel disease. J Crohns Colitis. (2021) 15:1019–31. 10.1093/ecco-jcc/jjaa252 33300552

[61] Li Q Wang C Tang C He Q Li N Li J. Dysbiosis of gut fungal microbiota is associated with mucosal inflammation in crohn's disease. J Clin Gastroenterol. (2014) 48:513–23. 10.1097/MCG.0000000000000035 24275714 PMC4059552

[62] Iliev ID. Mycobiota-host immune interactions in ibd: coming out of the shadows. Nat Rev Gastroenterol Hepatol. (2022) 19:91–2. 10.1038/s41575-021-00541-2 34815533 PMC9446421

[63] Hoffmann C Dollive S Grunberg S Chen J Li H Wu GD. Archaea and fungi of the human gut microbiome: correlations with diet and bacterial residents. PLoS ONE. (2013) 8:e66019. 10.1371/journal.pone.0066019 23799070 PMC3684604

[64] Lewis JD Chen EZ Baldassano RN Otley AR Griffiths AM Lee D. Inflammation, antibiotics, and diet as environmental stressors of the gut microbiome in pediatric crohn's disease. Cell Host Microbe. (2015) 18:489–500. 10.1016/j.chom.2015.09.008 26468751 PMC4633303

[65] Nguyen LH Örtqvist AK Cao Y Simon TG Roelstraete B Song M. Antibiotic use and the development of inflammatory bowel disease: a national case-control study in sweden. Lancet Gastroenterol Hepatol. (2020) 5:986–95. 10.1016/S2468-1253(20)30267-3 32818437 PMC8034612

[66] Samonis G Gikas A Anaissie EJ Vrenzos G Maraki S Tselentis Y. Prospective evaluation of effects of broad-spectrum antibiotics on gastrointestinal yeast colonization of humans. Antimicrob Agents Chemother. (1993) 37:51–3. 10.1128/AAC.37.1.51 8431017 PMC187603

[67] David LA Maurice CF Carmody RN Gootenberg DB Button JE Wolfe BE. Diet rapidly and reproducibly alters the human gut microbiome. Nature. (2014) 505:559–63. 10.1038/nature12820 24336217 PMC3957428

[68] Piovani D Danese S Peyrin-Biroulet L Nikolopoulos GK Lytras T Bonovas S. Environmental risk factors for inflammatory bowel diseases: an umbrella review of meta-analyses. Gastroenterology. (2019) 157:647–59.e4. 10.1053/j.gastro.2019.04.016 31014995

[69] Doron I Mesko M Li XV Kusakabe T Leonardi I Shaw DG. Mycobiota-induced iga antibodies regulate fungal commensalism in the gut and are dysregulated in crohn's disease. Nat Microbiol. (2021) 6:1493–504. 10.1038/s41564-021-00983-z 34811531 PMC8622360

[70] Zhernakova A Kurilshikov A Bonder MJ Tigchelaar EF Schirmer M Vatanen T. Population-based metagenomics analysis reveals markers for gut microbiome composition and diversity. Science. (2016) 352:565–9. 10.1126/science.aad3369 27126040 PMC5240844

[71] Jena A Dutta U Shah J Sharma V Prasad KK Shivaprakash RM. Oral fluconazole therapy in patients with active ulcerative colitis who have detectable candida in the stool: a double-blind randomized placebo-controlled trial. J Clin Gastroenterol. (2021) 56:705–11. 10.1097/MCG.0000000000001609 34516459

