# Supplementary material for: The ileal fungal microbiota is altered in Crohn's disease and is associated with the disease course
Source: Front Med (Lausanne). 2022 Sep 27;9:868812. doi: 10.3389/fmed.2022.868812 (PMC9551188; doi:10.3389/fmed.2022.868812)
Supplement: Supplementary file 1 [file Data_Sheet_1.pdf]

Supplementary Material

Supplementary Figure 1

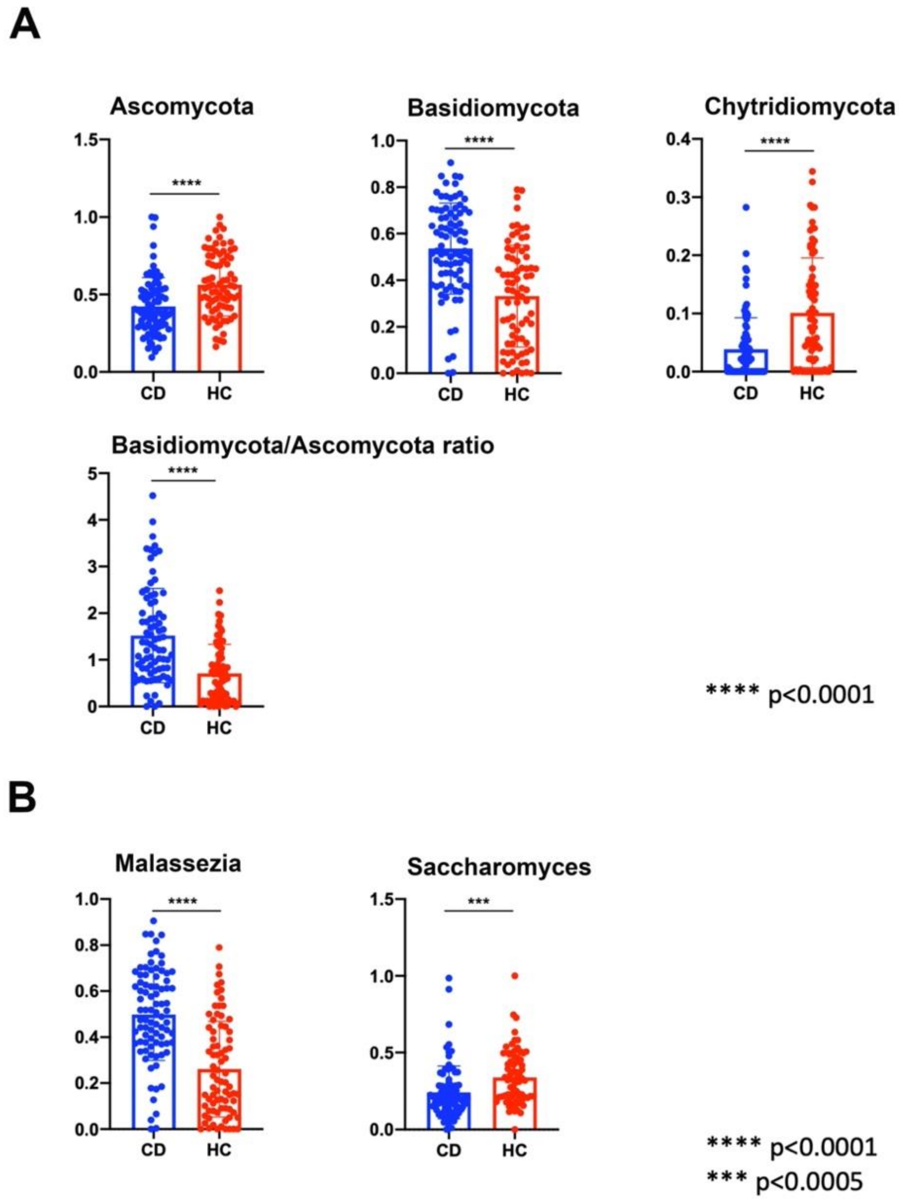

Supplementary Figure 1. Altered mucosa-associated mycobiota 44 Crohn's disease (CD) patients in comparison to 40 healthy controls (HC). (A) Abundances of fungal phyla and Basidiomycota/Ascomycota ratio, boxes coloured according to disease phenotype. Groups compared

using Independent Sample t-test. (B) Abundances of fungal genera, boxes coloured according to disease phenotype. Groups compared using Independent Sample t-test.

Supplementary Figure 2

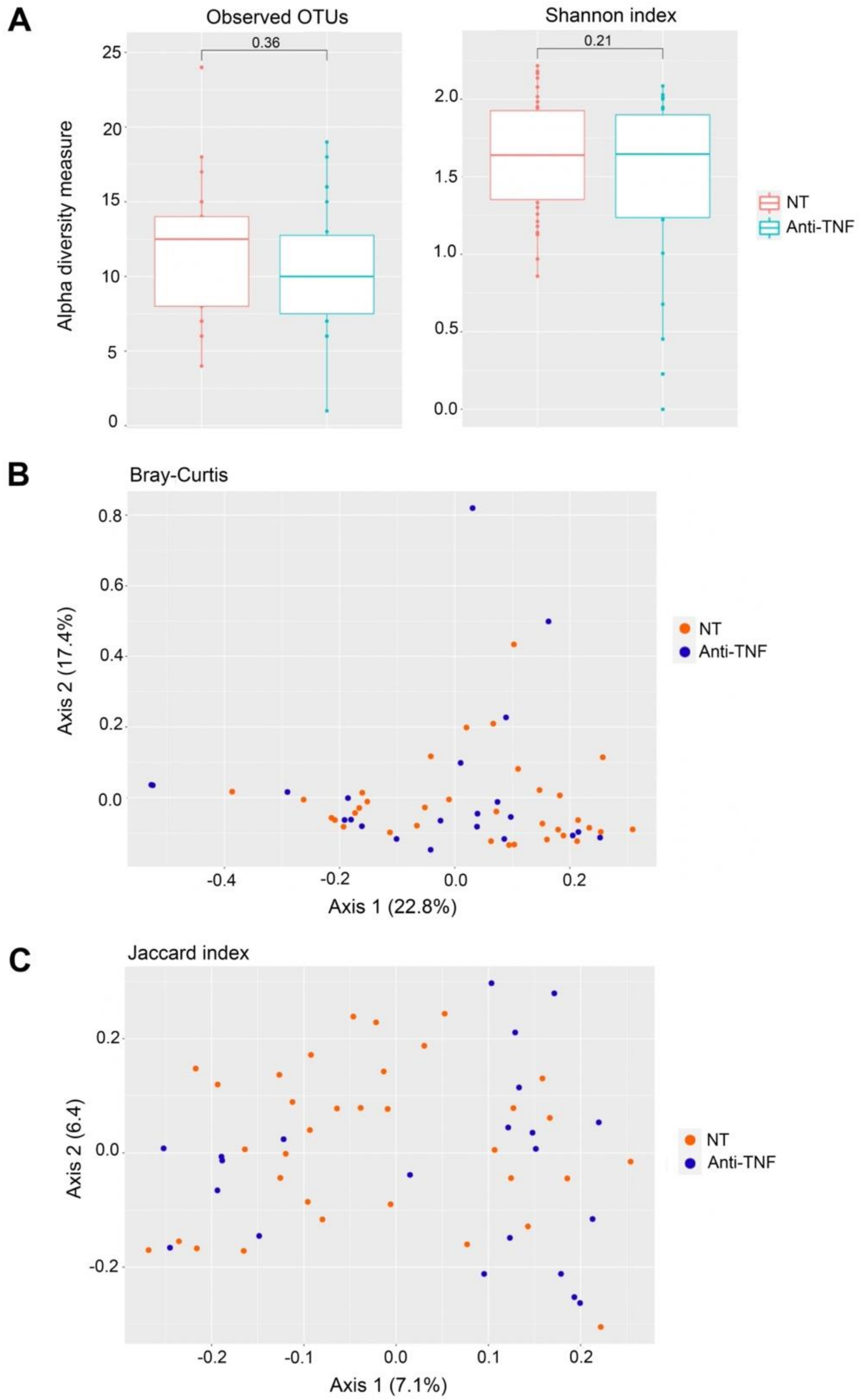

Supplementary Figure 2. Fungal mucosa-associated mycobiota in 11 Crohn's disease (CD) patients currently using anti-TNF agents (anti-TNF) compared to 18 CD patients with no medical treatment (NT). (A) Alpha diversity, according to observed operational taxonomic units (OTUs) (left) and Shannon index (right), boxplots coloured according to NT or anti-TNF. (B) Beta-diversity in anti-TNF and NT CD patients. Principal coordinates analysis of Bray-Curtis index with samples coloured according to anti-TNF and NT. The fraction of diversity captured by the coordinate is given in percentage on axes 1 and 2. (C) Beta-diversity. Principal coordinates analysis of Jaccard index with samples coloured according to TNF and NT. The fraction of diversity captured by the coordinate is given in percentage on axes 1 and 2.

Supplementary Figure 3

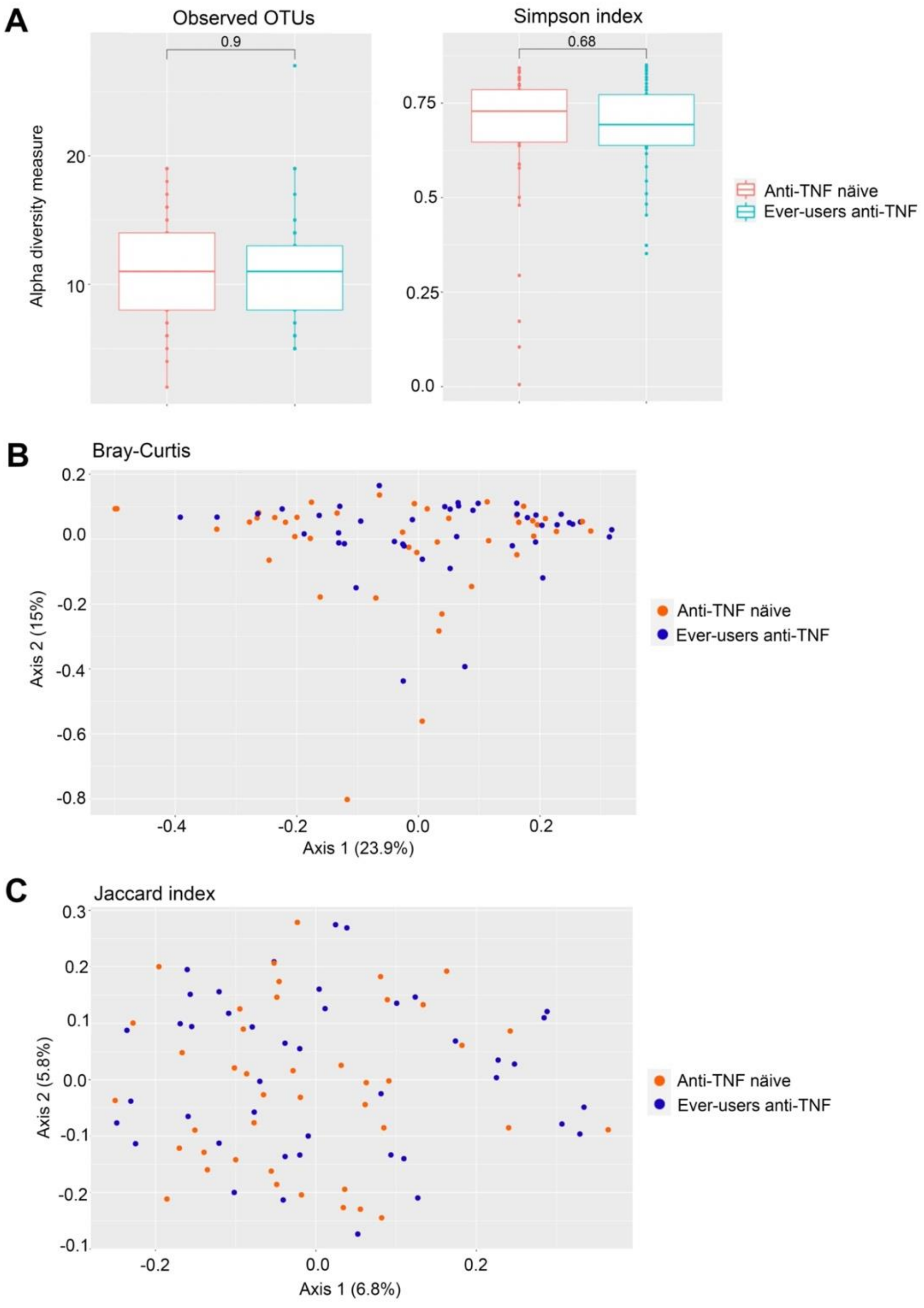

Supplementary Figure 3. Fungal mucosa-associated mycobiota in 23 anti-TNF naïve Crohn's disease (CD) patients (anti-TNF naïve) compared to 21 CD ever-users of anti-TNF treatment (Ever-users anti-TNF). (A) Alpha diversity, according to observed operational taxonomic units (OTUs) (left) and Shannon index (right), boxplots coloured according anti-TNF naïve or ever users of anti-TNF. (B) Beta-diversity in anti-TNF naïve CD patients. Principal coordinates analysis of Bray-Curtis index with samples coloured according to anti-TNF naïve and ever users of anti-TNF. The fraction of diversity captured by the coordinate is given in percentage on axes 1 and 2. (C) Beta-diversity. Principal coordinates analysis of Jaccard index with samples coloured according to anti-TNF naïve and ever-users of anti-TNF. The fraction of diversity captured by the coordinate is given in percentage on axes 1 and 2.

Supplementary Figure 4

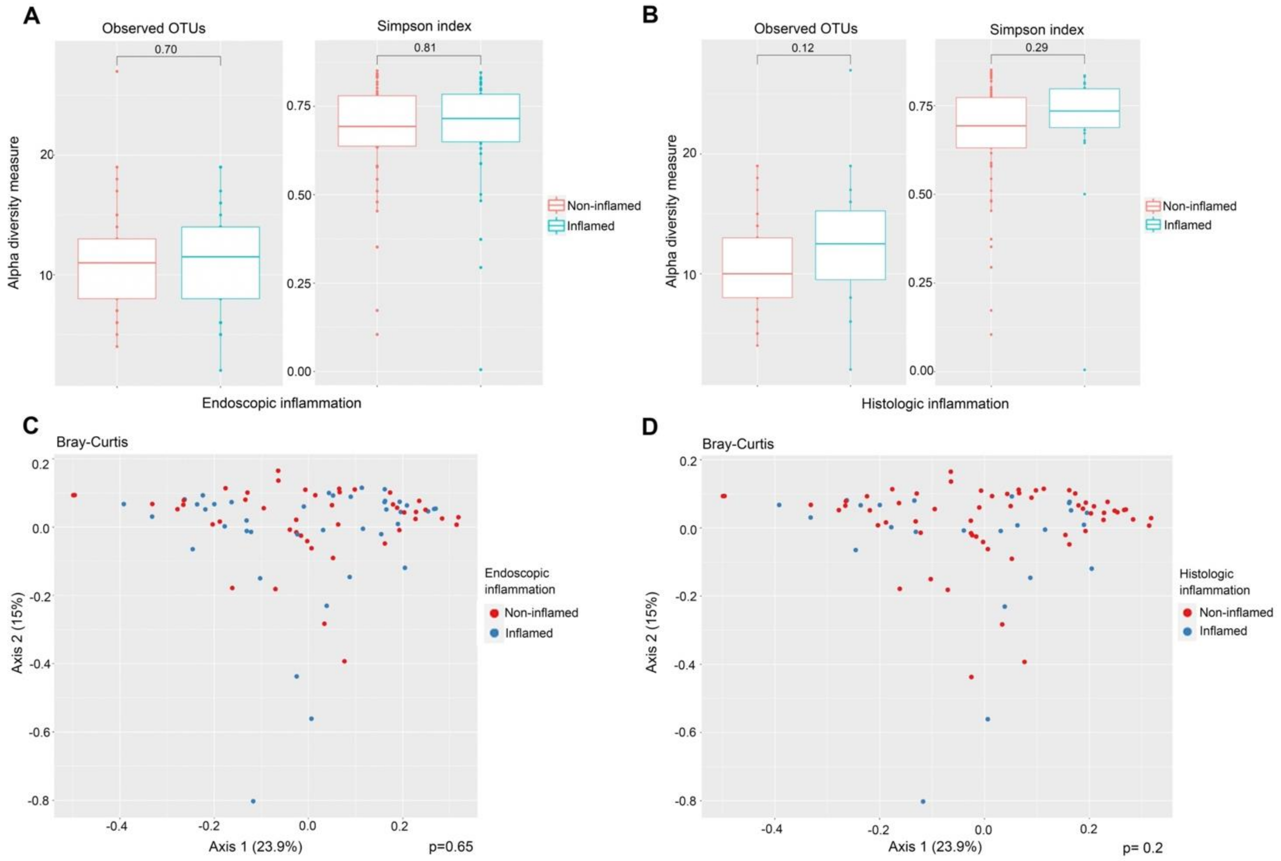

Supplementary Figure 4. Inflammation does not impact mucosa-associated mycobiota in CD patients overall (n=44). (A and B) Alpha-diversity, according to observed operational taxonomic units (OTUs) (left) and Simpson index (right), boxplots coloured according to endoscopic (A) or histologic (B) inflammation. (C and D) Beta-diversity. Principal coordinates analysis of Bray-Curtis dissimilarity with samples coloured according to endoscopic (C) or histological (D) inflammation. The fraction of diversity captured by the coordinate is given in percentage on axes 1 and 2. Groups compared using Permanova method.

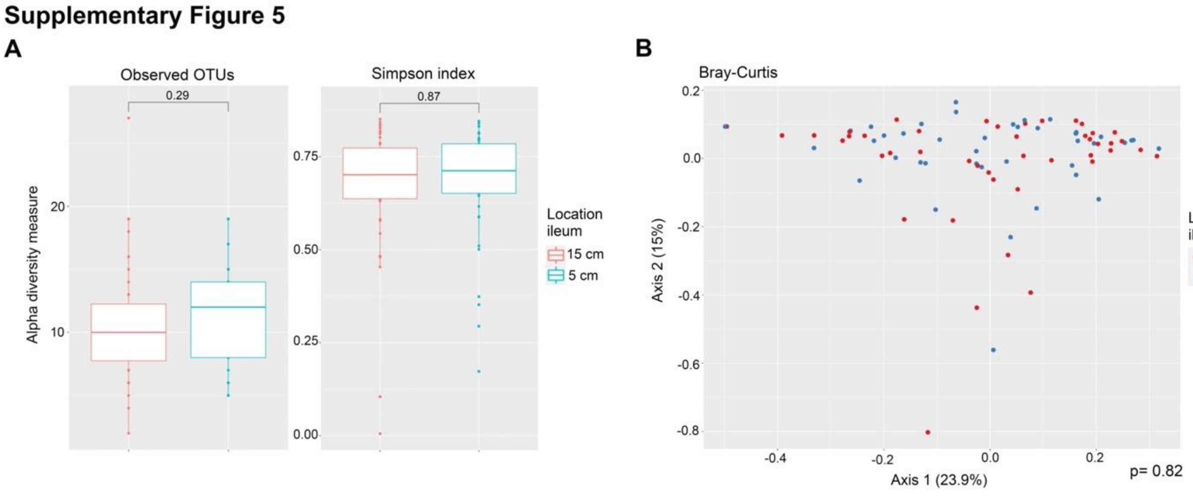

Supplementary Figure 5. Mucosal pinch biopsy location in the ileum of CD patients does not impact mucosa-associated mycobiota. 44 CD patients, biopsies sampled from 5- and 15-cm from the ileocecal valve or ileocolic anastomosis. (A) Alpha-diversity according to observed operational taxonomic units (OTUs) (left) and Simpson index (right), boxplots coloured according to biopsy location 5-cm or 15-cm. (B) Beta-diversity. Principal coordinates analysis of Bray-Curtis dissimilarity with sample colour according to biopsy location. The fraction of diversity captured by the coordinate is given in percentage on axes 1 and 2. Groups compared using Permanova method.

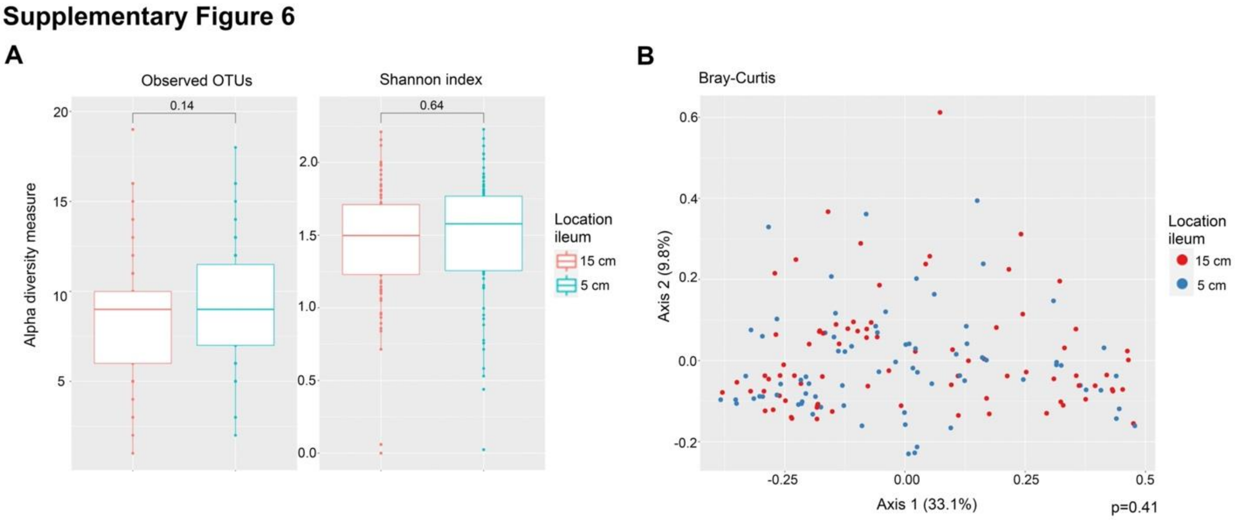

Supplementary Figure 6. Mucosa-associated mycobiota is similar across different ileal sub-locations in both Crohn's disease (CD) patients (n=44) and healthy controls (HC) (n=40). (A) Alpha-diversity according to observed operational taxonomic units (OTUs) (left) and Simpson index (right), boxplots coloured according to biopsy location 5-cm or 15-cm. (B) Beta-diversity. Principal coordinates analysis of Bray-Curtis dissimilarity with sample colour according to biopsy location. The fraction of diversity captured by the coordinate is given in percentage on axes 1 and 2. Groups compared using Permanova method.

Supplementary Table 1 Treatment escalation in Crohn's disease (CD) patients within one year after biopsy sampling.

**Treatment escalation**	**CD (n=44)**
CD patients with treatment escalation within one year	17 (38.6%)
CD patients without treatment escalation within one year	27 (61.4%)
**Type of treatment, *n (%)***	
Budesonide	1 (2.3%)
Glucocorticosteroids	6 (13.6%)
Azathioprine	3 (6.8%)
Anti-TNF treatment	5 (11.4%)
Surgery	2 (4.5%)
**Days until treatment escalation, mean (SD)**	135 (117)
